# Time-Dependent Color Stability of Three Nanohybrid Resin Composites After Cigarette Smoke Exposure: An In Vitro Study

**DOI:** 10.4317/jced.63645

**Published:** 2026-01-28

**Authors:** Hebert Iván Chipana-Chura, Cesar Juárez-Vizcarra, Fernando Mauricio Espada-Salgado

**Affiliations:** 1 School of dentistry, Faculty of Health Sciences, Private University of Tacna, Tacna, Peru

## Abstract

**Background:**

To determine whether cigarette smoke exposure increases time-dependent color change (E00) in nanohybrid resin composites.

**Material and Methods:**

This experimental, comparative, longitudinal in vitro study prepared 45 disc-shaped specimens, allocated to three groups (n = 15): Filtek Z350 XT, IPS Empress Direct, and Forma. Specimens were exposed daily to the smoke of six cigarettes for 28 days in a custom-built exposure chamber. Color was recorded at baseline and after 7, 14, 21, and 28 days using a VITA Easyshade spectrophotometer in the CIELAB color space. The primary outcome was color change calculated with the CIEDE2000 formula (E00). Perceptibility (0.8) and clinical acceptability (1.8) thresholds were considered. Repeated-measures ANOVA was used to evaluate the effect of time on E00.

**Results:**

E00 increased progressively over time for all three materials (main effect of time, p &lt; 0.001). Forma presented the lowest mean E values throughout follow-up, Filtek Z350 XT showed intermediate values, and IPS Empress Direct exhibited the highest color change. From day 14 onwards, all materials exceeded the perceptibility threshold and a relevant proportion of specimens surpassed the clinical acceptability threshold, with increasing magnitudes up to day 28.

**Conclusions:**

Daily exposure to cigarette smoke produced a progressive and statistically significant increase in color change (E00) in all three nanohybrid resin composites evaluated, confirming that exposure time directly influences chromatic stability.

## Introduction

Dental esthetics is a fundamental component of contemporary dentistry, as patients increasingly seek restorations that faithfully reproduce the optical characteristics of natural teeth (1). Among the many factors that can compromise these esthetic outcomes, tobacco-related staining is particularly relevant, especially in smokers whose continuous exposure to cigarette smoke can degrade both tooth structure and restorative materials (2 - 5). Although resin-based restorative materials have evolved considerably, including nanohybrid composites designed to optimize mechanical and optical performance, maintaining color stability in such challenging conditions remains difficult (1 , 6). Tobacco use continues to be a major global health problem: more than 1.3 billion people consume tobacco products, with substantial impact on both general and oral health (2 , 5). Nicotine, tar, and other smoke-derived components not only damage oral tissues but also induce color changes in esthetic restorations, to which composite resins are particularly susceptible because of the adsorption and penetration of smoke pigments (3 - 5). A color difference (E*ab) greater than 3.3 is considered clinically perceptible, whereas values between 1.0 and 2.0 typically require a trained observer (7 , 8). In smokers, repeated exposure accelerates esthetic deterioration and may reduce the clinical longevity of composite restorations (3 - 5 , 9). Nanohybrid resin composites were specifically developed to enhance mechanical properties, optical behavior, and color stability compared with earlier formulations (1 , 6). However, their performance under cigarette-smoke-induced pigmentation remains uncertain, as experimental studies and systematic reviews report variable degrees of discoloration depending on composite formulation, surface texture, finishing and polishing procedures, and exposure protocols (3 - 6 , 9 , 10). In light of these inconsistencies, the present in vitro study aimed to compare the color stability of three nanohybrid resin composites exposed to cigarette smoke, using standardized instrumental color measurements based on contemporary color-difference thresholds (7 , 8). Additionally, we evaluated whether the observed color changes exceeded the clinical perceptibility threshold, providing decision-relevant evidence for clinicians involved in esthetic restorative dentistry.

## Material and Methods

**Figure 1:**
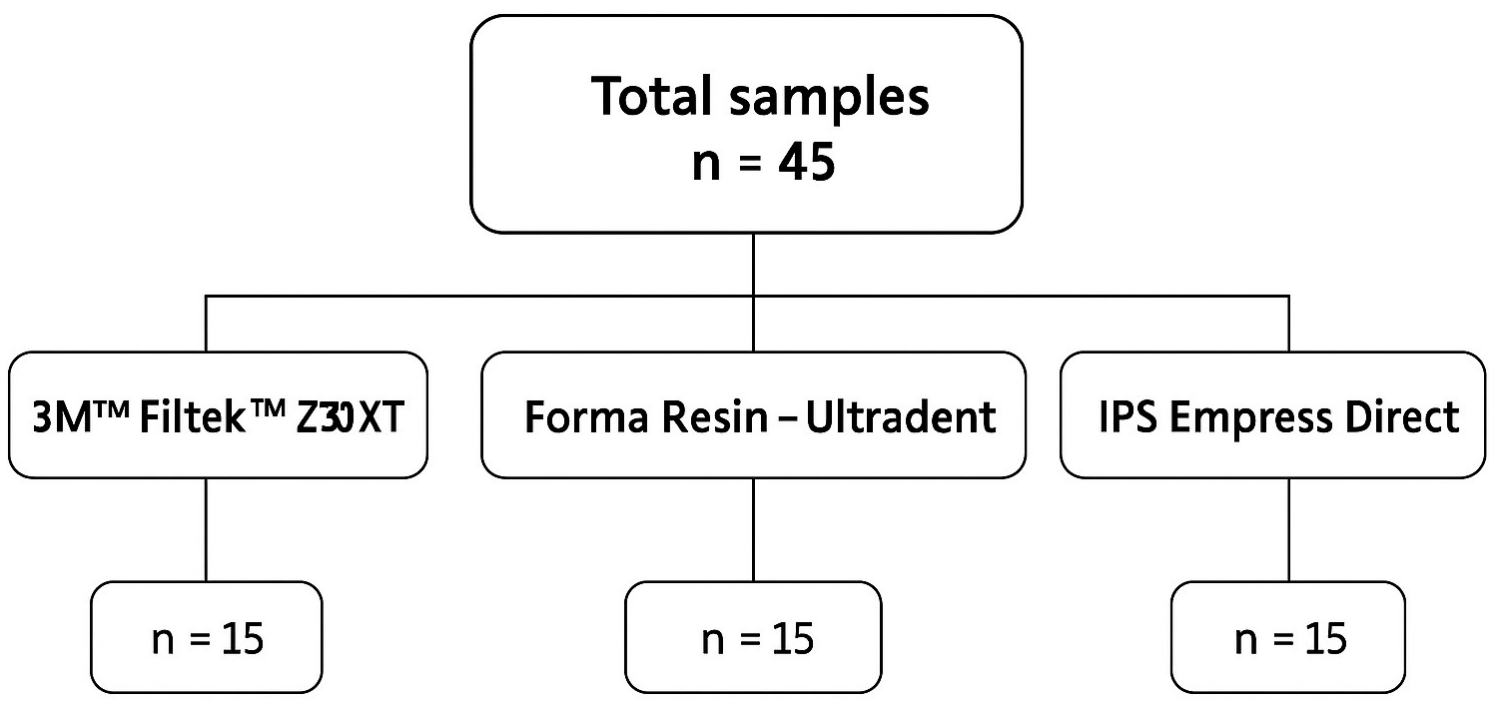
Distribution of specimens among the study groups.

- Study design and ethics This was a controlled, experimental, in vitro, comparative study with longitudinal follow-up. Color was measured at baseline and weekly during 28 days of exposure to cigarette smoke. Ethical approval was obtained under protocol FACSA-CEI/018-04-2025 (April 1, 2025). - Sample size and groups Sample size was calculated using G*Power software with a repeated-measures ANOVA, considering a moderate effect size (f = 0.2526; partial ² = 0.06), a significance level of 0.05, and a statistical power of 95.96%. The total sample consisted of 45 specimens, (Fig. 1) - Eligibility criteria Inclusion: photocured and polished discs that met the predefined dimensions.

1

Exclusion: surface defects, contamination, or products approaching their expiration date. Specimen preparation Forty-five discs were fabricated using a polyethylene mold (8 mm × 2 mm) in shade A1 (VITA). The composite was inserted, covered with a polyester (Mylar) strip, and pressed with a glass slide to obtain a flat surface before light curing. Finishing and polishing procedures were standardized. The specifications of the materials are summarized in Table 1.

Table 1

- Photopolymerization and polishing Specimens were light-cured with a VALO LED unit for 20 s at a nominal irradiance of 1000 mW/cm², allowed to rest for 5 min, and then stored for 24 h in an incubator at 37 °C. Polishing was performed with Sof-Lex discs in sequential order (medium, fine, superfine) for 10 s per disc at low speed. Afterwards, specimens were rinsed, ultrasonically cleaned when required, and stored in distilled water until baseline color measurement. - Smoke-exposure protocol A custom airtight chamber was used to provide daily exposure to the smoke of six conventional cigarettes per specimen for 28 consecutive days, under fixed flow and temperature conditions. Each cigarette was subjected to nine 2-s puffs separated by 60-s intervals. Discs were positioned equidistant from the smoke inlet. Between exposure sessions, specimens were kept in distilled water at 37 °C, renewed daily. - Color measurement Color was measured with a VITA Easyshade spectrophotometer (VITA Zahnfabrik, Bad Säckingen, Germany) in the CIE L*a*b* color space, using a D65 standard illuminant and a 2° observer. Baseline (day 0) and follow-up measurements (days 7, 14, 21, and 28) were recorded three times per specimen, and mean values were used for analysis. The primary color difference metric was CIEDE2000 (E00), computed from the L*, a*, and b* coordinates because it offers better perceptual uniformity and clinically supported thresholds for perceptibility (E00 = 0.8) and acceptability (E00 = 1.8) (7 , 8). Classic CIELAB color differences (E*ab) were also calculated descriptively to facilitate comparison with earlier literature that adopted the E*ab = 3.3 perceptibility threshold (7 , 8). - Randomization and blinding Specimens were randomly allocated to the study groups. The operator who recorded color measurements was blinded to group codes. - Outcomes Primary outcome: E00 over time and between materials. Secondary outcomes: proportion of specimens above EE00 = 0.8 and 1.8 at each time point; descriptive E*ab values. - Statistical analysis Normality and homogeneity of variances were verified before modeling. A two-way mixed repeated-measures ANOVA was applied (within-subject factor: time; between-subject factor: material). When the sphericity assumption was violated, the Greenhouse-Geisser correction was used. Pairwise comparisons were adjusted for multiplicity. Effect sizes were expressed as partial ² with 95% confidence intervals. The significance level was set at = 0.05.

## Results

This study evaluated the color stability of three nanohybrid resin composites exposed to cigarette smoke. CIEDE2000 color differences (E00) increased progressively over the 28-day exposure period for all materials. At all evaluation times, mean E00 values were highest for IPS Empress Direct, intermediate for 3M Filtek Z350 XT, and lowest for Forma Ultradent. Longer exposure times were associated with larger E00 values for each composite. The evolution of the CIE L* a* b* coordinates showed that cigarette smoke affected all three-color axes in each resin. A progressive decrease in L* was observed, indicating darkening, together with increases in b* (shift toward yellow) and in a* (shift toward red). These variations demonstrate a cumulative chromatic alteration over time and are detailed in Table 2.

Table 2

**Figure 2:**
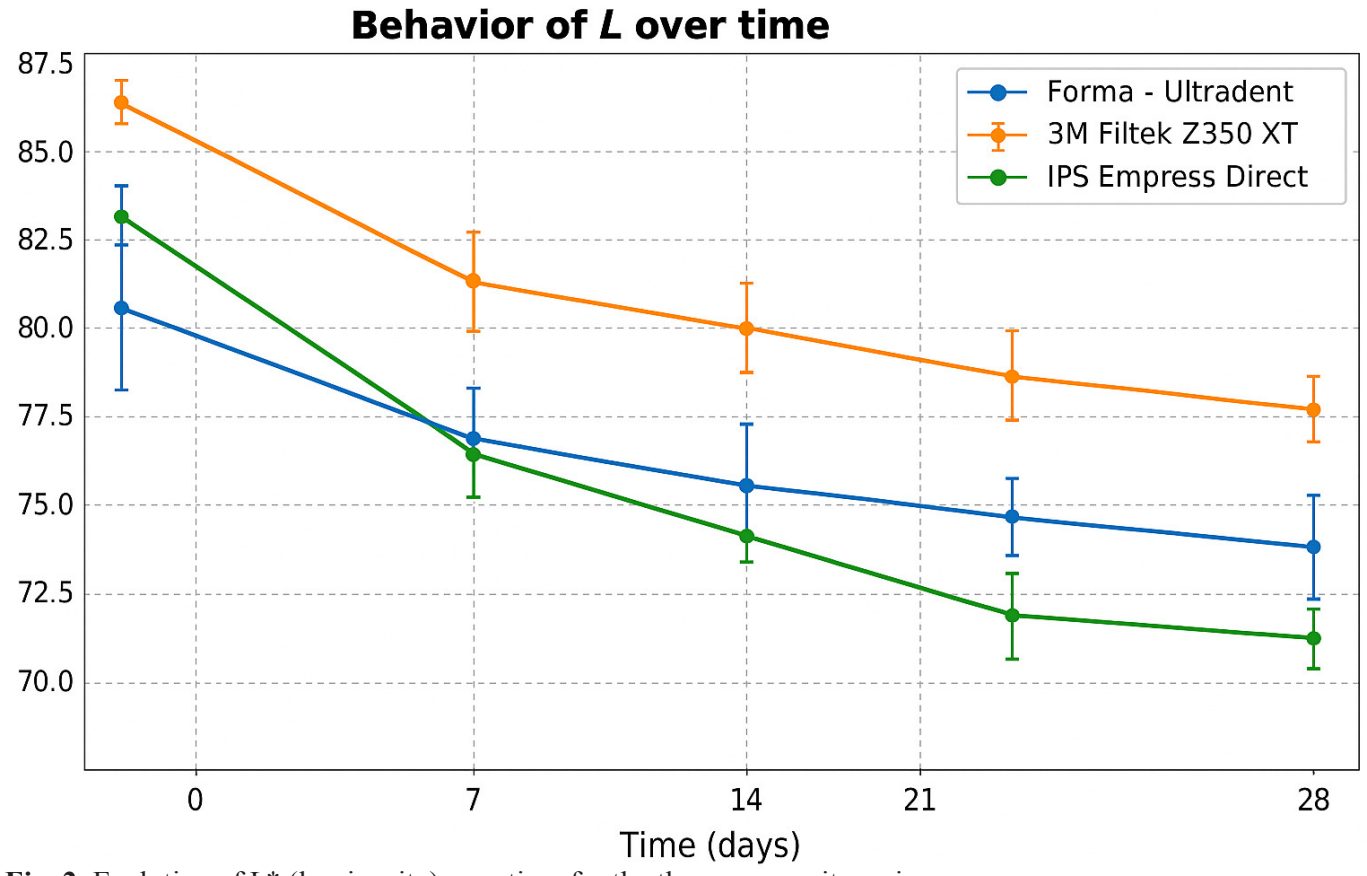
Evolution of L* (luminosity) over time for the three composite resins.

All three resins exceeded the classic CIELAB perceptibility threshold (E*ab &gt; 3.3) from day 7 onward. At day 28, IPS Empress Direct showed the greatest color change (E*ab = 10.27), followed by 3M Filtek Z350 XT (E*ab = 6.95) and Forma Ultradent (E*ab = 6.17). In the analysis of the L* coordinate (lightness), all resins showed a progressive decrease over the 28 days. The smallest reduction in L* was observed in Forma; Filtek Z350 XT showed an intermediate trend, and IPS Empress Direct showed the greatest darkening (Fig. 2).

2

**Figure 3:**
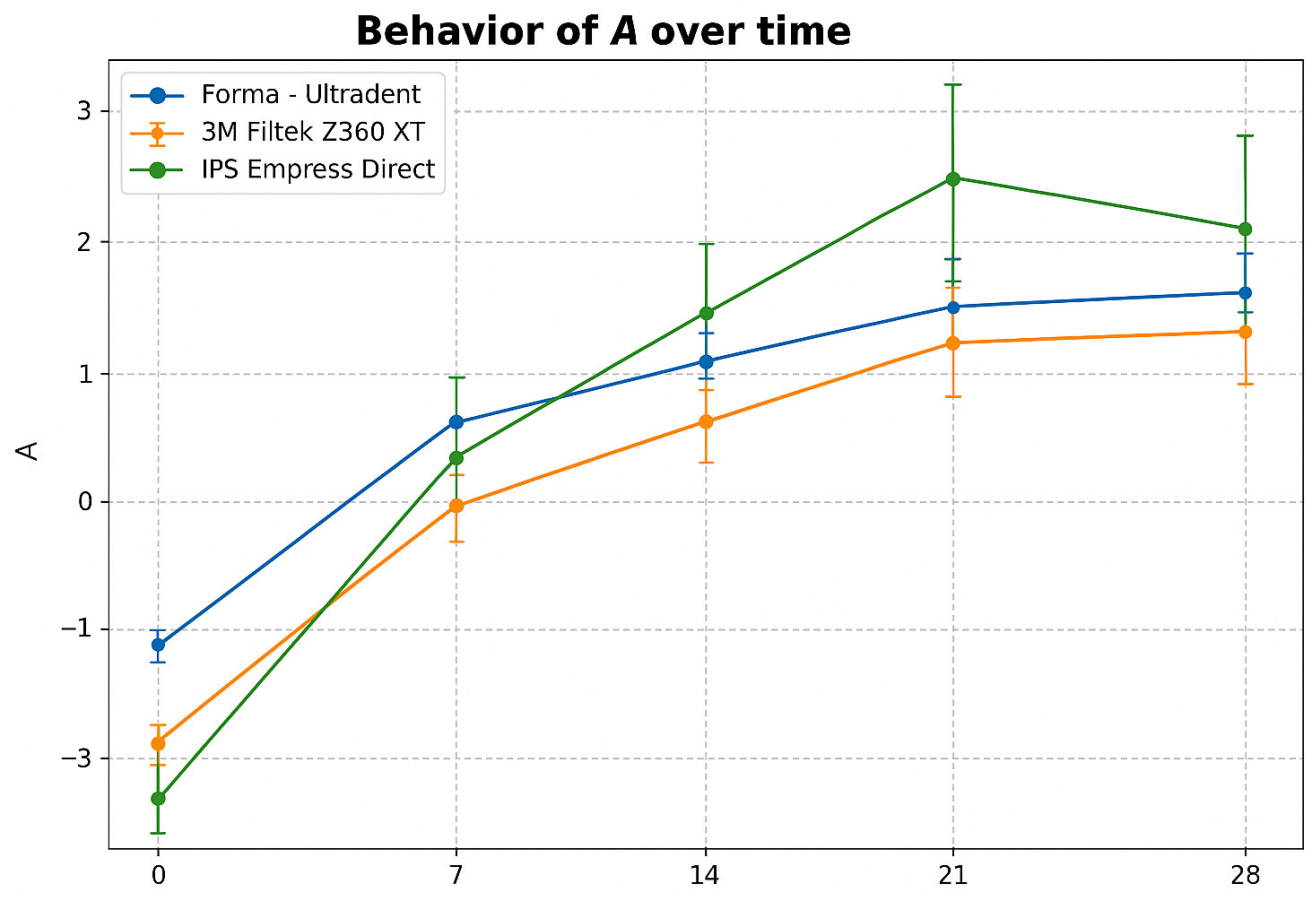
Evolution of a* (red/green) over time for the three composite resins.

At coordinate a* (red/green), all three resins showed a progressive increase in a* over time. At baseline, values were negative for all materials, with IPS Empress Direct showing the greatest shift toward green. As exposure progressed, measurements shifted toward the positive axis, indicating a tendency toward reddening. Regarding a* stability, Forma (Ultradent) showed the smallest change, Filtek Z350 XT (3M) an intermediate change, and IPS Empress Direct (Ivoclar) the largest (Fig. 3).

3

**Figure 4:**
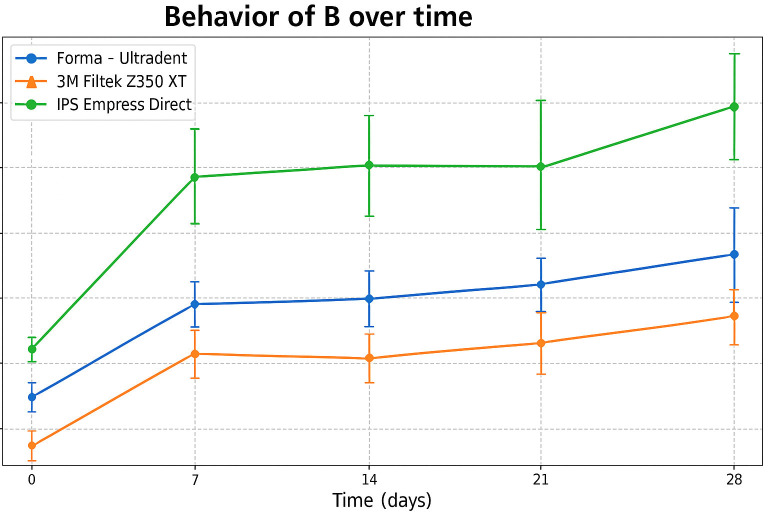
Evolution of b* (yellow/blue) over time for the three composite resins.

The b* coordinate (yellow/blue) showed a progressive increase in b* in all groups over the 28 days, indicating a tendency toward yellowing after exposure to smoke. The order remained consistent with the overall color change: Forma (Ultradent) showed the least shift, Filtek Z350 XT (3M) showed intermediate values, and IPS Empress Direct (Ivoclar) showed the greatest shift (Fig. 4).

4

Table 3 summarizes the evolution of color differences for each material. For clinical comparison, mean CIELAB color differences (E*ab) are presented, with corresponding mean E00 values in parentheses. Repeated-measures ANOVA performed on E00 showed significant within-group increases over time for all materials and significant between-group differences at all time points.

Table 3

## Discussion

This in vitro study showed a progressive increase in E00 for all three nanohybrid resin composites during 28 days of daily exposure to cigarette smoke. Repeated-measures analyses confirmed significant main effects of time and material, whereas the time × material interaction was not significant. From day 14 onwards, a large proportion of specimens in each material exceeded the perceptibility threshold (E00 &gt; 0.8), and an increasing subset also surpassed the clinical acceptability threshold (E00 &gt; 1.8), indicating clinically relevant changes according to CIEDE2000 criteria (7 , 8). Our findings are consistent with the synthesized evidence showing that tobacco and nicotine promote staining of dental tissues and restorative materials, with cumulative effects over time (9). Mechanistically, the deposition and adsorption of smoke condensates (e.g., tar, nicotine) on the surface produce measurable optical shifts even when the bulk structure remains stable. In addition, finishing and polishing modulate chromogen retention, with smoother surfaces tending to accumulate fewer pigments (1 , 6 , 9 , 10). In this context, the lower E00 values observed for Forma and the higher values for IPS Empress Direct are compatible with formulation-related and surface-response differences among nanohybrid composites. The material-dependent behavior observed in our study can also be interpreted in light of resin chemistry and polymer network formation. The consistent ranking of E00 values over time, with IPS Empress Direct showing the highest color changes, 3M Filtek Z350 XT intermediate values, and Forma Ultradent the lowest, suggests that IPS Empress Direct is more susceptible to smoke-induced discoloration, whereas Forma Ultradent exhibits comparatively greater color stability under the tested conditions. The selection and proportion of dimethacrylates (e.g., Bis-GMA, UDMA, Bis-EMA, TEGDMA) determine the degree of conversion, free volume, water affinity, and ultimately color stability; curing conditions further influence network development and its properties (11 - 16). Greater water sorption and hygroscopic expansion are associated with increased susceptibility to optical change and surface degradation, phenomena that may amplify extrinsic staining under smoke exposure (13 - 15). The inorganic phase also contributes to optical behavior and sorption pathways. Barium-containing glasses add radiopacity, but particle size/shape and filler loading can affect water uptake and optical stability (17 , 18). Zirconia-based systems may improve mechanical and surface performance, supporting greater polishing durability and favorable light-scattering characteristics (19). Composites with fluoride-releasing glass phases can undergo ionic exchange and surface alterations over time; such changes have been linked to greater color change and reduced microhardness under staining and brushing challenges, in line with increased chromogen retention (20 - 22). Consistently, different finishing and polishing systems have been shown to generate variable roughness levels, and smoother surfaces tend to exhibit significantly lower color change after exposure to staining agents, although often still above perceptibility and acceptability thresholds (23). The analysis of CIELAB coordinates in our data supports these mechanisms: L* decreased in all materials (darkening), while a* and b* increased, reflecting shifts toward reddish and yellowish hues, patterns already described for tobacco-related staining of resin-based materials (8). Clinical implications. Given the sustained global burden of tobacco use (2), clinicians should anticipate color changes that are perceptible and, frequently, clinically unacceptable in resin composites exposed to smoke. Material selection, meticulous finishing/polishing, and maintenance protocols (including periodic re-polishing) may help mitigate extrinsic staining and extend esthetic longevity (1 , 6 , 9 , 10). It is advisable to educate smoking patients about stain accumulation and realistic maintenance expectations (8). Strengths and limitations. Strengths of this study include a controlled exposure model, standardized instrumental colorimetry, and interpretation based on E00 thresholds (7 , 8). However, inherent limitations of in vitro designs remain (absence of saliva, biofilm, abrasion, and the variability of real-world smoking patterns). In addition, no non-smoke control group was included, which limits our ability to separate the effects of smoke exposure from those of water storage alone. Future studies should evaluate combined effects of smoke and hygiene regimens and compare broader classes of composites under simulated clinical conditions (1 , 6 , 11).

## Conclusions

Cigarette smoke exposure produced a progressive and statistically significant increase in CIEDE2000 color differences (E00) for all three nanohybrid resin composites, indicating that exposure time directly influences chromatic stability. Forma Ultradent showed the greatest color stability over the 28-day period, maintaining the lowest E00 values at all time points. 3M Filtek Z350 XT exhibited an intermediate behavior, whereas IPS Empress Direct was the most susceptible to smoke-induced pigmentation, with the highest E00 values throughout the experimental period under the tested conditions.

## Figures and Tables

**Table 1:** Specifications of the nanohybrid resin composites.

Composite	Lot	Color	Manufacturer	Type	Organic Matrix	Inorganic filler (% wt./% vol)	Particle size
Filtek™ Z350 XT	10080845	A1	3M ESPE, USA	Nanohybrid	Bis-GMAUDMATEGDMAPEGDMABis-EMA	Non-agglomerated silica 20 nm and zirconia 4–11 nm; aggregated silica/zirconia clustered filler; total inorganic filler ≈ 78.5% wt (≈ 63.3% vol)	0.6 – 20 µm
IPS Empress® Direct	Z0771L	A1	Ivoclar Vivadent AG	Nanohybrid	Bis-GMAUDMADCP	Inorganic fillers 52–59% vol; barium glass; mixed Si-Zr oxide; barium–aluminum fluor-silicate glass	0.03 µm – 16.3 µm
Forma Ultradent	D0Q77	A1	Ultradent Products, Inc.	Nanohybrid	Bis-GMAUDMATEDMABis-EMA	Zirconia/silica base, barium glass, and radiopaque ytterbium trifluoride; overall filler load ≈ 64.8%	5 - 50nm

Notes: wt = weight; vol = volume; particle sizes as reported by manufacturers. Abbreviations: Bis-GMA, UDMA, TEGDMA, PEGDMA, Bis-EMA, DCP.

**Table 2:** Mean L*, a*, b*, ΔE*ab and ΔE00 values according to resin and exposure time.

Resin	Days	L*	a*	b*	ΔE*ab	ΔE00
Forma Ultradent	0	81.74	- 1.21	16.35	-	-
	7	78.26	0.43	19.07	3.5	0.76
	14	77.21	0.95	19.13	4.41	0.64
	21	75.97	1.49	19.92	5.53	0.65
	28	75.32	1.63	21.02	6.17	0.69
3M Filtek Z350 XT	0	86.53	- 1.88	14.38	-	-
	7	82.91	- 0.31	16.97	3.57	0.75
	14	81.59	0.46	16.62	4.74	0.6
	21	79.55	1.13	17.37	6.31	0.84
	28	78.73	1.25	18.38	6.95	0.82
IPS Empress Direct Ivoclar	0	83.39	- 2.43	16.81	-	-
	7	77.24	0.14	24.81	6.11	1.23
	14	74.77	1.40	24.91	8.06	1.54
	21	72.34	2.63	24.75	10.06	2.03
	28	72.05	2.28	26.74	10.27	1.93

L* = lightness; a* = red–green chromatic coordinate (positive values indicate a shift toward red, negative toward green);b* = yellow–blue chromatic coordinate (positive values indicate a shift toward yellow, negative toward blue);ΔE*ab = color difference in the CIELAB space; ΔE00 = color difference calculated with the CIEDE2000 formula.

**Table 3:** Post hoc comparisons between and within groups.

Resin	T1 (Day 7)	T2 (Day 14)	T3 (Day 21)	T4 (Day 28)	Within-group differences
	Mean (M)	Mean (M)	Mean (M)	Mean (M)	
(A) Forma Ultradent	3.5 (0.76) Aa	4.41 (0.64) Ab	5.53 (0.65) Ac	6.17 (0.69) Ad	0.001***
(B) 3M Filtek Z350 XT	3.57 (0.75) Aa	4.74 (0.6) Ab	6.31 (0.84) Ac	6.95 (0.82) Ad	0.001***
(C) IPS Empress Direct	6.11 (1.23) Ba	8.06 (1.54) Bb	10.06 (2.03) Bc	10.27 (1.93) Bc	0.001***
Differences between groups	0.001*	0.001*	0.001*	0.001*	

Notes:Values are mean (M) CIELAB color differences (ΔE*ab); corresponding mean CIEDE2000 values (ΔE00) are shown in parentheses. Inferential statistics (p values) are based on ΔE00. Uppercase superscripts (A, B, C) indicate between-group comparisons for each column; values with different letters in the same column differ significantly (one-way ANOVA, post hoc test). Lowercase superscripts (a, b, c, d) indicate within-group comparisons across time in each row (repeated-measures ANOVA). *p value for between-group differences at each time point (one-way ANOVA). ***p value for within-group differences over time (repeated-measures ANOVA).

## Data Availability

The datasets used and/or analyzed during the current study are available from the corresponding author.
